# Knowledge, attitudes, and practices towards drug-food interactions among patients at public hospitals in eThekwini, KwaZulu-Natal, South Africa

**DOI:** 10.4314/ahs.v22i1.79

**Published:** 2022-03

**Authors:** Emmanuella C Osuala, Boikhutso Tlou, Elizabeth B Ojewole

**Affiliations:** 1 Discipline of Pharmaceutical Sciences, School of Health Sciences, College of Health Sciences, University of KwaZulu-Natal, P B X54001 Durban 4000 South Africa; 2 Department of Public Health, School of Nursing & Public Health, University of KwaZulu-Natal, P B X54001 Durban 4000 South Africa

**Keywords:** Drug-food interactions, patients, knowledge, attitudes, practices

## Abstract

**Background:**

Drug-food interactions can lead to adverse drug reactions and therapy failure which can potentially impact patient safety and therapy outcome.

**Objectives:**

This study assessed patients' knowledge, attitudes and practices regarding drug-food interactions.

**Methods:**

A cross-sectional study was conducted among patients at three public hospitals in eThekwini, KwaZulu-Natal. Statistical analysis was performed using SPSS® version 25. The association between demographic variables and patients' knowledge, attitudes and practices were assessed.

**Results:**

Of the 342 patients, 70.5% were female, and the mean age was 42.87±0.89 years. Almost 50% of patients had secondary level education, and 64% were unemployed. About 52% of patients had high knowledge of drug-food interactions; however, only 30–50% of the patients could identify potential drug-food interactions of their drugs. More than half of the patients (51.5%) answered that they took multivitamin pills with medications and 61.7% responded they consulted healthcare professionals for drug-food interactions' information before taking new medications. Few patients (15.2%) had experienced drug-food interactions.

**Conclusions:**

Overall, patients had gaps in their knowledge and practices, and positive attitudes towards drug-food interactions. Many patients could not identify food items that can potentially interact with their drugs. It is important that education and medication counselling are provided to patients to prevent drug-food interactions, ensure optimal drug therapy and patient safety.

## Introduction

Drug-food interactions (DFI) can result in adverse drug reactions (ADR) or therapy failure, which can impact patients' safety.^1^ Food and its components can alter the effects of drugs by interfering with pharmacodynamics and pharmacokinetic processes resulting in decreased drug efficacy or increased drug toxicity.^2^,^3^ The nutritional status of the patients may also be affected, as drugs can alter the body's ability to utilize particular food or nutrient.^4^ For instance, the interaction between the anticoagulant warfarin and foods rich in vitamin K has been reported to cause poor anticoagulation outcomes in patients.^5^,^6^ Also, concomitant ingestion of antibiotics such as tetracycline and ciprofloxacin, with milk and dairy products can result in decreased absorption of the antibiotics.^7^ Such an effect can lead to antibiotic resistance in infections that are only moderately susceptible to antibiotics. Thus, it is indispensable to ensure that the patients' knowledge, attitudes, and practices (KAP) are adequate to prevent DFIs.

The potential risk for DFIs have been determined in different countries with varying rates ranging from approximately 20–89%.^1^,^8^–^11^ Although this prevalence has not been determined in South Africa, the reported high prevalence imposes a need for careful attention, especially among susceptible patients. Elderly patients, patients taking multiple medications, those with chronic conditions such as diabetes, hypertension, or hyperlipidaemia and patients with nutritional deficiencies are at an increased risk for DFIs.^3^,^12^ Exposure to DFI can also result from patients' lack of awareness of these interactions. 1 Therefore, knowledge of DFI by patients is essential to prevent adverse events and ensure the success of drug treatment.

Many outpatients take one or more prescription drugs in combination with over-the-counter drugs. Such combinations may increase the chance of DFI and potential toxicity.^13^ As patients play an integral role in their healthcare and safety, accurate information and advice from healthcare professionals would ensure that patients are well informed about the medicines they take.^14^ According to the Joint Commission Standard in the United States, patients are to be educated on the safe and efficacious use of their medicines and potential DFIs.^15^ To ensure that the goals of drug safety and efficacy are met, it is essential to understand the level of patients' KAP regarding drug-food interaction.

Previous studies on DFI knowledge of patients have reported low knowledge among the patients.^16^,^17^ Findings from a study in France showed that only 40% of patients were aware of warfarin-food interactions.18 However, little is known about the KAP of patients as regards to DFI in sub-Saharan Africa. More so, in the South African healthcare setting particularly in KwaZulu-Natal, there is a paucity of information on the patients' KAP towards DFI. Therefore, this study assessed KAP of patients regarding DFI at public hospitals in eThekwini district, KwaZulu-Natal, and determined the potential factors associated with patients' KAP.

## Methods

### Ethics Approval

This study received an approval from the University of KwaZulu-Natal Biomedical Research Ethics Committee (UKZN BREC; BE 371/18) and the KwaZulu-Natal Department of Health (KZ_201808_027).

### Study design and setting

This was a cross-sectional study among patients in medical outpatient pharmacy department at three public hospitals in eThekwini district. KwaZulu-Natal is one of South Africa's most populous provinces with approximately 10.3 million people and is divided into 11 health districts. The eThekwini district is in Kwa-Zulu-Natal that is located on the east coast of South Africa and its population accounts for approximately one-third of the provincial population.^19^

The convenience sampling was used to select the study hospitals from a list of hospitals in the eThekwini district. 20 Patients waiting at the medical outpatient pharmacy were selected using systematic random sampling to participate in this study. Eligible participants were patients aged 18 years or older, and willing to participate in the study. The sample size for this study was determined using the Glen D Israel formula which is a method for determining the minimum sample size required for a survey.^21^ The sample size was 400, considering the total number of patients (>20,000) that visit the three hospitals monthly, a 95% confidence interval and a 5% margin of error.

### Questionnaire and data collection procedure

A questionnaire was utilised to obtain information on the patient's KAP towards DFI. The questionnaire was developed from themes reported in the literature.^1^,^12^,^22^ The questionnaire contained dichotomous and multiple-choice questions and consisted of four sections: Iincluded questions on the socio-demographics of the patients, disease conditions and alcohol intake, II- had questions on general knowledge of DFI, patient's medication intake, food intake and knowledge of selected drug-food combinations that could lead to interactions, III- consisted of questions on the patient's attitudes towards DFI, and IV- included questions on practices towards preventing DFI, the patients' sources of information about DFI, experience of DFI, and the experience of ADR due to DFI. An overall score was calculated for each section. Each correct/positive answer was given a maximum score of one and each wrong/negative answer was scored zero. Thus, the maximum overall score for the knowledge, attitude and practice questions were 10, 8 and 8 respectively. The questionnaire was piloted among 20 patients to ensure validity and clarity of questions and was completed within an average time of 20 minutes. The questionnaire was translated to isiZulu language which is one of the predominant languages in the district.

The patients were approached while seated at the medical outpatient pharmacy, and were selected using systematic random sampling. Every fifth patient was approached to participate in the study and if unwilling to participate, the next patient was approached. Patients who were willing to participate were given the information sheet and consent form. Patients who gave consent were then handed the questionnaire to complete.

### Data analysis

The statistical package for social sciences (SPSS®) version 25 was used for data analysis. Continuous variables were expressed as means and standard error of the mean, while categorical variables were expressed as frequencies and percentages. The association between the categorical variables was determined using the chi-square test or Fisher's exact test. Binary logistics regression was executed to determine factors associated with the patient's KAP regarding DFIs. Results are presented as odds ratios and their respective confidence intervals at 95%. In all analyses, a p-value of ≤ 0.05 was considered statistically significant.

## Results

### Patients' demographic and clinical characteristics

A total of 342 patients completed the questionnaire, giving a response rate of 85.5%. Most patients were female (n=241, 70.5%), and the mean age was 42.87±0.89 years. As shown in Table 1, few patients (34.5%) reported having ≥3 disease conditions and the most commonly reported disease condition was hypertension (n=144, 42.1%).

**Table 1:** Demographic and clinical characteristics of the patients

Characteristic	Frequency (n)	Percentage (%)
**Gender (N=342)**		
Male	101	29.5
Female	241	70.5
**Ethnic group (N=342)**		
African (Blacks)	221	64.6
Indian	65	19.0
White	29	8.5
Colored	27	7.9
**Age category (N=332)**		
29 years and below	83	24.3
30 – 39 years	76	22.2
40 – 49 years	49	14.3
50 – 59 years	59	17.3
60 years and above	65	19.0
**Educational level (N=340)**		
Primary	29	8.5
Secondary	182	53.2
Tertiary	120	35.1
Uneducated	9	2.6
**Employment status (N=342)**		
Employed	82	24.0
Self-employed	41	12.0
Unemployed	219	64.0
**Occupation (N=141)**		
Employee	68	19.9
Business	22	6.4
Retired	22	6.4
Housewives	10	2.9
Students	8	2.3
Others	11	3.2
**Marital status (N=340)**		
Single	185	54.1
Married	108	31.6
Divorced/Separated	13	3.8
Widowed	34	9.9
**Locality (N=312)**		
Urban	301	88.0
Rural	11	3.2
**Disease conditions**		
Hypertension	144	42.1
Hyperlipidaemia	68	19.9
Diabetes	86	25.1
Muscle pain/spasm	59	17.3
Arthritis	52	15.2
**Number of disease conditions per** **patient (N=331)**		
1	122	35.7
2	91	26.6
≥3	118	34.5
**Alcohol intake (N=339)**		
Yes	77	22.5
No	262	76.6

### Patients' knowledge of drug-food interactions

As shown in Table 2, most patients knew that some drinks can interfere with the effectiveness of drugs (n=285, 83.3%) and that drugs can alter a person's nutritional status (n=266, 77.8%). Few patients could identify that grapefruit juice interacts with over 45 different medicines (n=94, 27.5%). The mean knowledge score of the patients was 5.78±0.10. Almost half of the patients (n=166, 48.5%) were reported to have low knowledge of DFIs.

**Table 2:** Knowledge responses of the patients regarding drug-food interactions

Knowledge questions	Correct response		Incorrect response		Missing response	
	
	n	(%)	n	(%)	n	(%)
Some foods can interfere with the effectiveness of drugs in the body	226	66.1	111	32.5	5	1.4
Some drinks can interfere with the effectiveness of drugs in the body	285	83.3	51	14.9	6	1.8
Some foods can increase or decrease the action of a drug	234	68.4	100	29.2	8	2.4
Some drugs can alter the nutritional status of a patient	266	77.8	70	20.5	6	1.8
Knowledge of factors that can influence drug-food interactions*	132	38.6	189	55.3	21	6.1
Drug-food interactions can occur when drugs interact with diet, iron/vitamin supplements, alcohol and fruit juices*	126	36.8	186	54.4	30	8.8
Foods most likely to cause drug-food interactions*	110	32.2	170	49.7	62	18.1
Fruit and its juice that can interact with over 45 different medicines (grapefruit juice)	94	27.5	163	47.6	85	24.9
Juice to be avoided when taking anticoagulants like warfarin (cranberry)	58	17.0	206	60.2	78	22.8
Drink to avoid when taking most medicines (alcohol)	285	83.3	43	12.6		

* Multiple choice questions- No. of patients who identified all correct options for the questions with more than one correct response

Table 3 reports on patients who correctly identified potential DFIs of their medications. The patients' knowledge was generally low as 30.2%–50.0% could identify potential DFIs.

**Table 3:** Patients that correctly identified potential drug-food interactions of their medications

Medications	Foods	*Patients taking medication n, (%)	Patients taking medication and food n, (%)	Patients taking medication that correctly identified the interactions n, (%)
**Warfarin**	Leafy green vegetables such as spinach, kale broccoli	54 (15.8)	47 (87.0)	27 (50.0)
**Simvastatin**	Grapefruit juice	81 (23.7)	21 (25.9)	29 (35.8)
**Enalapril**	Bananas, sweet potato, avocados	119 (34.8)	75 (63.0)	36 (30.2)
**Ciprofloxacin**	Dairy products such as milk	48 (14.0)	31 (64.6)	21 (43.8)
**Levothyroxine**	Fibre- rich foods cabbage, cauliflower, millet	27 (7.9)	22 (81.5)	10 (37.0)
**Metronidazole**	Alcoholic drinks	43 (12.6)	8 (18.6)	20 (46.5)
**Isoniazid**	Chicken liver, tuna, mackerel	10 (2.9)	8 (80.0)	5 (50.0)
**Glimepiride**	Alcoholic drinks	51 (14.9)	8 (15.7)	23 (45.1)
**Theophylline**	Caffeine rich foods such as coffee, tea, chocolate	21 (6.1)	11 (52.4)	7 (33.3)

* Total number of patients who participated in the survey N= 342 (represented for column 3)

### Attitude of the patients towards drug-food interactions

The mean attitude score of the patients was 6.54±0.08 out of an overall score of eight. Most patients (n=207, 60.5%) had a positive attitude towards DFIs. As shown in Table 4, majority of the patients thought the timing of taking drugs with respect to meals is important for treatment (n=318, 93.0%), DFIs can be prevented (n=275, 80.4%) and that it is important to report DFIs to healthcare professionals (n=319, 93.2%). Only a few patients reported that they were well informed regarding DFIs (n=167, 48.8%).

**Table 4:** Patients' responses to attitude-related questions

Attitude question	Yes		No		*Total responses	
	n	(%)	n	(%)	n	(%)
Do you think that drug-food interactions have any effect on treatment?	263	76.9	76	22.2	339	99.1
Do you think that the timing of taking drugs with respect to meals is important for treatment?	318	93.0	23	6.7	341	99.7
Do you think some foods should be avoided when taking certain drugs?	283	82.7	57	16.7	340	99.4
Do you think drug-food interactions can be prevented?	275	80.4	60	17.5	335	98.0
Do you think information regarding drug-food interactions should be provided by the healthcare professional?	311	90.9	27	7.9	338	98.8
Do you think you are well informed about drug-food interactions?	167	48.8	172	50.3	339	99.1
Are you interested in learning more about drug-food interactions?	300	87.7	41	12.0	341	99.7
Is it important to report drug-food interactions to your healthcare professional?	319	93.2	22	6.4	341	99.7

* The total number of responses may not add up to 342, due to missing values

Yes- was the positive/correct response

### Patients' practices regarding prevention of drugfood interactions

Overall, 53.8% (n=184) of the patients were reported to have poor practices. The mean practice score of the participants was 6.25±0.07 out of eight. Of the patients (n=52, 15.2%) that had experienced DFIs, only 20 patients (5.8%) reported the DFIs to healthcare professionals. Moreover, of the patients (n= 45, 13.2%) who experienced ADR due to DFIs, only few (n=17, 4.97%) reported the ADR to healthcare professionals. Table 5 shows the patients' responses to practice-related questions. While most patients responded correctly to the questions, some of them (n=97, 28.4%) reported taking their medications using other beverages like fruit juice, tea, coffee, and alcohol as compared to water alone. Half of the patients (n=176, 51.5%) reported taking vitamin pills concurrently with medication, and 37.7% (n=129) did not consult healthcare professionals for information regarding DFIs before taking new medications. Most patients (n=234, 68.4%), commonly sourced DFI information from healthcare professionals, however, several patients (n=109, 31.9%) sourced from the internet.

**Table 5:** Responses of patients to practice-related questions

Practice questions	Correct response		Incorrect response		*Total responses	
	n	(%)	n	(%)	n	(%)
Which of the following do you use in taking your medications? (water alone)	245	71.6	97	28.4	342	100.0
Do you stir your medications into food?	313	91.5	26	7.6	339	99.1
Do you take your vitamin pills at the same time as your medications?	176	51.5	159	46.4	335	97.9
Before taking your medication do you read the prescription label on the container?	313	91.5	27	7.9	340	99.4
Before taking your medication do you read the patient information leaflet?	283	82.7	58	17.0	341	99.7
Do you consult your healthcare professional for information regarding drug-food interactions before taking any new medication?	211	61.7	129	37.7	340	99.4
Do you follow the recommendation for the timing of taking drugs with respect to meals?	311	90.9	30	8.8	341	99.7
Do you follow the recommendation of foods to avoid when taking your medications?	302	88.3	37	10.8	339	99.1

* The total responses may not add up to 342, due to missing values

### Factors associated with patients' knowledge, attitudes, and practices towards drug-food interactions

The patient's knowledge and practices in the univariate analysis were not significantly associated with the demographic variables, however, the patient's attitudes were significantly associated with no alcohol intake (p=0.004). Logistic regression analysis also showed a significant association between patient's attitudes and no alcohol intake (p=0.006). As shown in Table 6, patients who did not take alcohol were more than twice likely to demonstrate positive attitude as compared to those who take alcohol.

**Table 6:** Factors associated with attitudes of patients using logistic regression

Variable	Attitude score	
	OR (95%CI)	p-value
**Gender**		
Male	-	-
Female	1.187 (0.702–2.006)	0.522
**Age**	0.994 (0.979–1.010)	0.459
**No. of disease conditions per patient**		
1	-	-
2	1.348 (0.752–2.416)	0.316
≥ 3	1.586 (0.893–2.818)	0.116
**Alcohol Intake**		
Yes	-	-
No	2.254 (1.268–4.005)	0.006**

** Significant association at p-value≤0.01

Variables with a p-value of 0.2 or less in the chi-square analysis were included in this regression model. These were gender and alcohol intake.

Age and number of disease conditions were included in the model.

## Discussion

This study assessed the KAP of patients regarding DFIs, and further assessed the DFI knowledge of some selected drugs taken by patients. About half of the patients had a low level of knowledge regarding DFIs which could be due to poor education on DFIs. Many studies have reported low level of knowledge regarding drug safety among patients.^23^–^25^ In studies that reported on patients' knwledge of anticoagulant therapy, questions about DFIs were incorrectly answered.^16^–^18^ Similarly, our study findings showed low level of knowledge of DFIs.

The majority of the patients correctly answered that alcohol should be avoided with most medications. It is known that alcohol should be avoided when taking most medicines,^12^ and this information could have been received by the patients which could have led to their correct answers. Few patients (27.5%) correctly identified that grapefruit juice could interact with a lot of medicines, while 17.0% patients identified that cranberry juice could interact with anticoagulants such as warfarin. Patients who do not take drugs that interact with grapefruit and those that do not take anticoagulants may not be aware of foods that may interact, and this may have influenced their knowledge. About 30% of patients taking drugs such as enalapril, simvastatin, levothyroxine, and theophylline knew that potassium-rich foods, grapefruit juice, fibre-rich foods, and caffeine could interact with their medicines. Potassium containing foods and salt substitutes can interact with ACE inhibitors such as enalapril precipitating the risk of hyperkalaemia. 26 Grapefruit juice increases blood levels of simvastatin by about 260% if taken concurrently,^27^ therefore, taking them concurrently should be avoided because of potential for adverse effects.^28^,^29^ Also taking levothyroxine with foods rich in dietary fibre can result in sub therapeutic effect, and taking theophylline with caffeine can lead to adverse effects.^29^, ^30^ These findings raise concerns about the patients' risk of potential DFIs ensuing from their inadequate knowledge. Patients should be provided with information on DFI, the timing of drug administration, drug interactions and appropriate use of drugs to ensure drug safety and optimal therapeutic outcome.

The attitude of the patients was generally positive, although half of the patients thought they were not well informed about DFIs and most indicated interest in learning more. The patients appear to be right that they were not well-informed regarding DFIs as this corresponded with their low knowledge scores. Our results contrast with those of other studies in which patients' perception of being well informed about their medications compared poorly with their actual tested knowledge. 32,33 There is a low level of knowledge about DFIs among patients, therefore, adequate counselling and education regarding DFI should be provided for these patients.

This study identified poor practices that may predispose patients to DFIs. Few patients reported the use of alcohol, fruit juice, tea, and coffee in taking their medications. These beverages can interact with drugs, and water should be used to take medicines except otherwise instructed by the healthcare professional.^1^ Patients reported taking their multivitamins concurrently with their drugs. Multivitamins are regarded as food and thought to be safe, however, co-administration of drugs with multivitamins could lead to interactions.^12^ The patients' poor practices may lead to ADR due to DFIs.

The patients mostly obtained DFI information from their healthcare professionals. Similar to previous studies, healthcare professionals are often regarded as highly preferred sources of information.^32^,^34^ According to the Wellcome Global Monitor report on South Africa, 74% of the public (n=1000) indicated that they trust doctors and nurses the most for medical advice.^35^ Despite reporting that healthcare professionals were a source of information, some patients did not seek information on DFI from healthcare professionals before taking new drugs. This practice is of serious concern because of the danger of interactions associated with over-thecounter drugs.^14^ This reinforces the need for adequate counselling and education of the patients, and emphasises the importance of patients communicating with healthcare professionals regarding all drugs they take.

A few patients reported experiencing ADRs caused by DFIs and majority did not report the occurrence of ADRs to healthcare professionals. Patients mostly reported experiencing interactions from drug and food combinations such as alcohol and antiretroviral drugs, and milk and ciprofloxacin. Interaction of antiretroviral drugs with alcohol could precipitate liver toxicity among human immunodeficiency virus (HIV) infected patients, and impair drug metabolism that can lead to drug-resistance.^36^ This is of particular concern in healthcare setting where there is a high burden of HIV. Therefore, increased patient education and behavioural intervention are necessary to avert potential ADRs.

The influence of demographic characteristics such as age, educational status and living circumstances on patients' knowledge and practices towards DFIs have been reported in previous studies.^1^,^16^ Unlike these studies, no demographic characteristic was significantly associated with the knowledge and practices of patients in our study. However, there was a significant association between no alcohol intake and positive attitudes towards DFI. It is possible that patients who do not take alcohol are more health-conscious and thus have better attitudes towards DFIs. Overall, intervention efforts are required to improve patients' knowledge regarding DFIs and preventing potential ADRs caused by DFIs.

## Conclusion

This study showed that patients had low knowledge of DFIs and in identifying food types that could potentially cause interactions between drugs and foods. The study findings are useful to address the limited literature in the area of patients' KAP regarding DFIs. Our study findings could have been limited by the type of questions asked, particularly the dichotomous questions used in establishing the patients' attitudes. Moreover, responses to the questions were self-reported and may have been subject to recall bias. However, the study presents important data on KAP regarding DFIs among patients at public hospitals in eThekwini, KwaZulu-Natal. Future studies among inpatients at hospitals in other districts and other South African provinces may be conducted in order to improve patients' KAP regarding DFIs. Patients' education and counselling regarding DFIs should be emphasized, interactive educational DFI resources that will inform patients about preventing and reducing possible risks of DFIs should be developed.
